# Double outlet right ventricle presenting in an adult woman: a case report

**DOI:** 10.1016/j.radcr.2022.02.026

**Published:** 2022-03-02

**Authors:** Dian Komala Dewi, Derry Priadhi Perdhana

**Affiliations:** Department of Radiology, Faculty of Medicine Universitas Padjadjaran, Jl. Pasteur No. 38, Pasteur, Kec. Sukajadi, Kota Bandung, Jawa Barat, Bandung, 40161, Indonesia

**Keywords:** Cardiac magnetic resonance imaging, Congenital cardiac malformation, Double outlet right ventricle

## Abstract

Double outlet right ventricle (DORV) is a congenital cardiac malformation that occurs in 1%-3% of individuals with congenital heart defects. Cardiac magnetic resonance imaging (MRI) may play an important role in the anatomy of the ventricular septal defect, functional status of both ventricles, and in identifying any residual stenosis or regurgitation or coexistent anomalies. Here, we present a case of a 28-years-old woman who came to our emergency department with shortness of breath. The patient felt shortness of breath on exertion and improved with rest. Clinical examination showed no abnormalities except low oxygen saturation of 65%. After echocardiography and cardiac MRI were done, it was concluded that she had a double outlet right ventricle with peri membranous ventricular septal defect (VSD), pulmonary hypertension, and pericardial effusion. This study highlights the role of cardiac MRI in assessing DORV.

## Introduction

Double outlet right ventricle is a type of abnormal ventriculoarterial connection in which both large arteries are fully or partially connected to the right ventricle, this definition implying that more than 50% of each great vessel arises from the right ventricle, which is known as ``the 50% rule.''[1], [2], [3], [4] Radiological examination is essential to localize and characterize the type of congenital malformation. In this report, we present an adult woman with a double outlet right ventricle who presented with dyspnea on exertion; together with the echocardiography and cardiac MRI findings.

## Case presentation

**Fig. 1:**
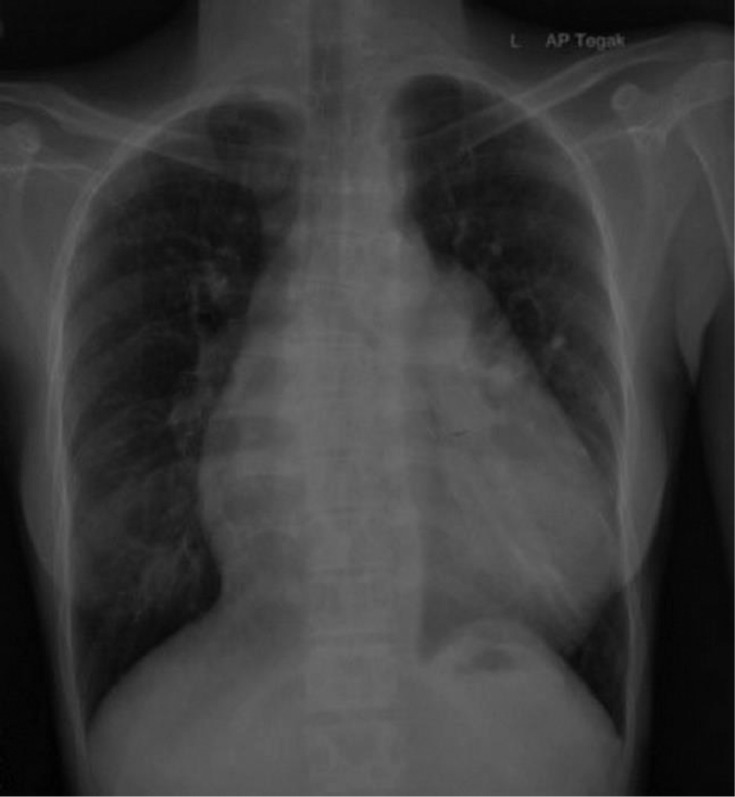
A chest radiograph revealed cardiomegaly without signs of pulmonary congestion, right bronchopneumonia.

**Fig. 2:**
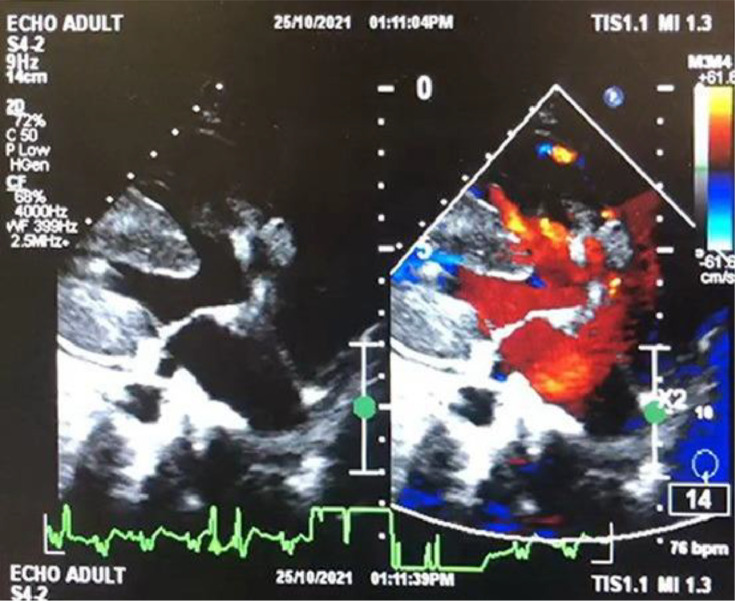
Echocardiography revealed a dilated LA, dilated RA, biventricular hypertrophy, PM VSD 16-19 mm.

**Fig. 3(A):**
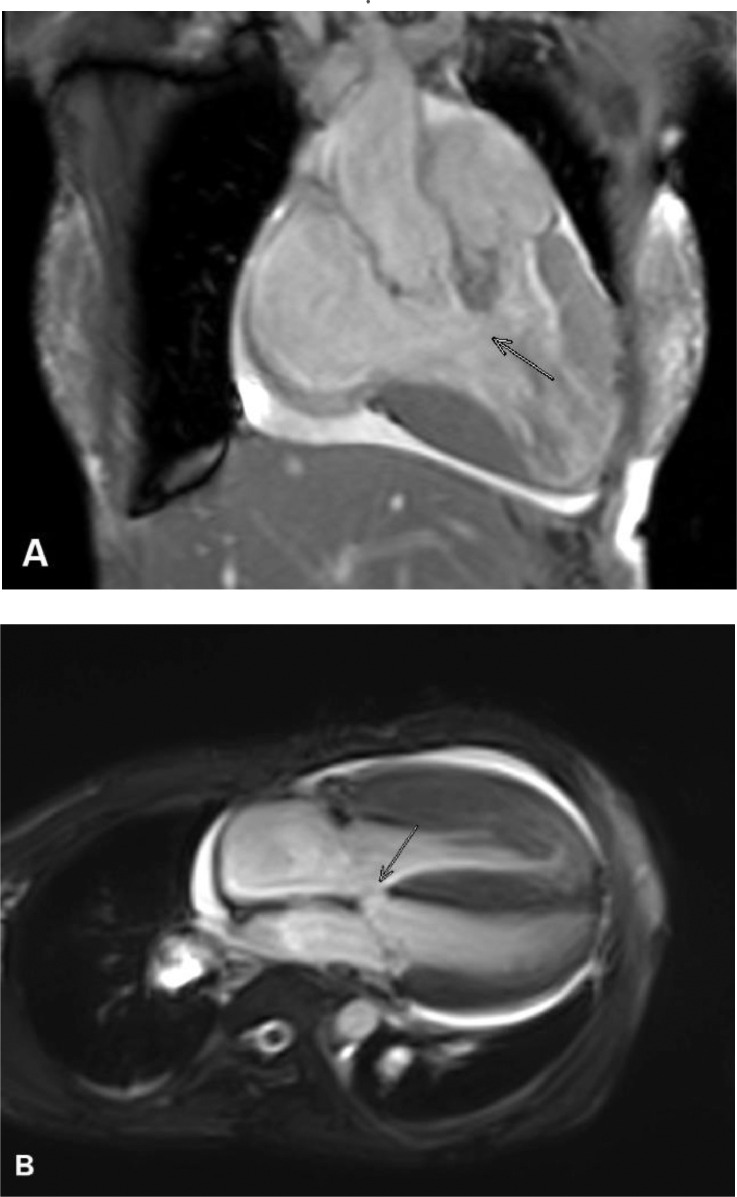
Double outlet right ventricle (white arrow), (B) ventricular septal defect (white arrow).

**Fig. 4(A):**
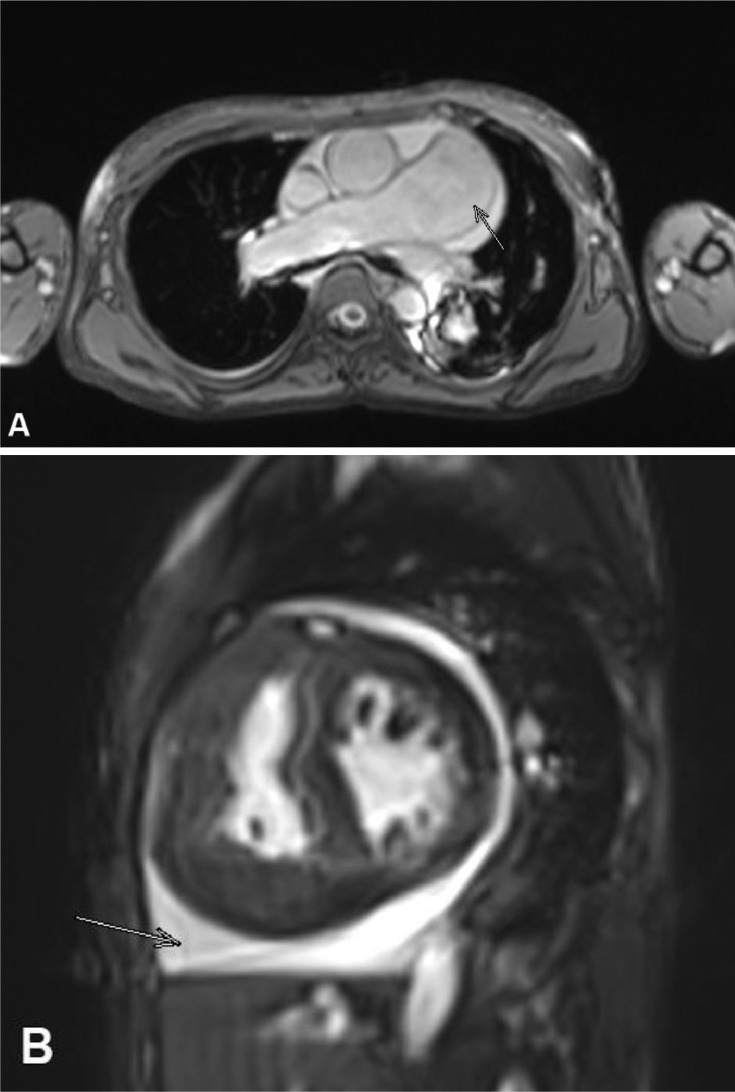
Pulmonary hypertension (white arrow), (B) pericardial effusion (white arrow).

A 28-year-old woman presented to our emergency department with shortness of breath. The patient felt shortness of breath on exertion and improved with rest. Shortness of breath did not subside with changes in position such as tilt to the left or right. Shortness of breath is not accompanied by fever, cough, and wheezing. The patient had no night sweats, loss of appetite, weight loss, vomiting, chest pain, and hypertension. The clinical examinations showed no abnormality except for low oxygen saturation 65%. Routine laboratory examinations were performed and the results were within normal limits. A frontal chest x-ray revealed cardiomegaly without signs of pulmonary congestion, right bronchopneumonia (Fig. 1), which prompted further investigation with echocardiography. Echocardiography revealed a dilated left atrium (LA), dilated right atrium (RA), biventricular hypertrophy, perimembranous (PM) VSD 16-19 mm, bidirectional shunt mainly right to left with overriding aorta >50% (Trans VSD gradient 2-3 mmHg) (Fig. 2). Dilated and confluence pulmonary artery (PA), main pulmonary artery (MPA) diameter 36 mm, right pulmonary artery (RPA) diameter 20 mm, left pulmonary artery (LPA) diameter 16 mm. Cardiac MRI revealed a double outlet right ventricle with ventricular septal defect, pulmonary hypertension, and moderate pericardium effusion (Fig. 3(A), Fig. 4(A)).

The patient was advised to undergo surgery, but due to personal reasons, the patient refused to do so. The patient was discharged and advised to do routine check-ups with the cardiologist.

## Discussion

Double outlet right ventricle (DORV) is a congenital cardiac malformation involving the right ventricular outflow tract (RVOT). Based on the consensus of the Congenital Heart Surgery Nomenclature and Database Project, DORV is defined as a ventriculoarterial relationship in which both the aorta and the pulmonary artery drain mostly into the RV.[5]

DORV can occur as a single condition or in combination with other cardiac or noncardiac anomalies. This condition is rare and accounts for only 1%-1.5% of all congenital heart disease.[4,6] Its reported incidence varies from 0.03 to 0.14 cases per 1000 live births.[4] In the United States, the incidence of DORV reaches 0.09 cases per 1000 live births.[6] Other anomalies that can be seen with DORV include coarctation of the aorta, hypoplasia of the aortic arch, or interrupted aortic arch. DORV is a common finding in hearts with right atrial isomerism. Several chromosomal abnormalities such as trisomy 13, trisomy 18, and the deletion of chromosome 22q11 are also associated with DORV.[1,4]

DORV can be classified according to the location of the VSD to the large arteries (aorta and pulmonary arteries). Based on this classification, Lev et al. divide DORV into 4 types, namely DORV with subaortic, sub pulmonary, doubly committed, and non-committed/remote VSDs. The advantage of this classification system is that it is simple and able to distinguish each type of DORV. However, this classification system alone is not sufficient to determine the management of DORV.[4] In some rare cases of DORV, an intact intraventricular septum may be found.[7]

The diagnosis of DORV is an indication for surgery. Examinations that need to be done before surgery include echocardiography, cardiac MRI, or cardiac catheterization. Cardiac MRI can provide information regarding intracardiac anatomy, the aortic arch, pulmonary artery morphology, 3-dimensional relationships between the heart chambers and great vessels, and right and left ventricular function.[4]

The result of the chest radiograph and cardiac MRI of the patient suggested that there was a double outlet right ventricle with peri membranous ventricular septal defect (VSD), pulmonary hypertension, and pericardial effusion.

## Conclusion

DORV is a complex form of congenital cardiac malformation. The diagnosis of DORV is an indication for surgery, therefore examinations need to be done before surgery. Radiology has an important role in the diagnosis of DORV. Radiological examinations such as cardiac MRI provides accurate anatomic information in patients with DORV, which aids in pre-operative planning as well as follow-up planning.

## Patient consent

Patient consent was obtained for the article.
